# Reducing Flow Table Update Costs in Software-Defined Networking

**DOI:** 10.3390/s23239375

**Published:** 2023-11-23

**Authors:** Wen Wang, Lin Yang, Xiongjun Yang, Jingchao Wang

**Affiliations:** Academy of Military Sciences, Beijing 100141, China; yfyer@163.com (X.Y.); wangjc.2000@tsinghua.org.cn (J.W.)

**Keywords:** software-defined networking, flow table update, rule dependency

## Abstract

In software-defined networking (SDN), the traffic forwarding delay highly depends on the latency associated with updating the forwarding rules in flow tables. With the increase in fine-grained flow control requirements, due to the flexible control capabilities of SDN, more rules are being inserted and removed from flow tables. Moreover, the matching fields of these rules might overlap since multiple control domains might generate different rules for similar flows. This overlap implies dependency relationships among the rules, imposing various restrictions on forwarding entries during updates, e.g., by following update orders or storing entries at specified locations, especially in flow tables implemented using ternary content addressable memory (TCAM); otherwise, mismatching or packet dropping will occur. It usually takes a while to resolve and maintain dependencies during updates, which hinders high forwarding efficiency. To reduce the delay associated with updating dependent rules, in this paper, we propose an updating algorithm for TCAM-based flow tables. We formulate the TCAM maintenance process as an NP-hard problem and analyze the inefficiency of existing moving approaches. To solve the problem, we propose an optimal moving chain for single rule updates and provide theoretical proof for its minimum moving steps. For multiple rules arriving at a switch simultaneously, we designed a dynamic approach to update concurrent entries; it is able to update multiple rules heuristically within a restricted TCAM region. As the update efficiency concerns dependencies among rules, we evaluate our flow table by updating algorithms with different dependency complexities. The results show that our approach achieves about 6% fewer moving steps than existing approaches. The advantage is more pronounced when the flow table is heavily utilized and rules have longer dependency chains.

## 1. Introduction

The cloud–edge computing paradigm presents great challenges for networking technology tasked with serving millions of endpoints, each with various communication requirements, e.g., low delay, high bandwidth, security, etc. First, computing services are distributed into the cloud or multiple edges, creating more communication demands between the core cloud and edge clouds. Thus, switches have to maintain increasing numbers of forwarding entries in flow tables to forward traffic to the correct service. Second, due to the mobility characteristics of endpoints (e.g., sensors, unmanned vehicles), the communication quality between the endpoints and edge clouds varies, depending on the changing environment. Forwarding entries are added or deleted in flow tables as connections go up or down. In particular, in highly dynamic environments, these changes may occur at a rate of approximately hundreds to thousands of entries per second [1,2]. Thus, the flow table update efficiency is quite critical to ensure the forwarding quality.

In the context of software-defined networking (SDN), new entries are inserted into corresponding flow tables at the arrival of a new flow, so that switches are able to look up flow tables to match the flow and find the forwarding actions. The flow entry update is usually in the order of milliseconds in a flow table [3], while the packet lookup rate is in the order of millions of packets per second. Slow flow entry updates will lead to packets being buffered in switches and will result in the dropping of packets in cases of buffer overflow. As the flow table update delay is relatively slow when compared to packet lookup, it is paramount to speed up flow table updates to avoid the packet-forwarding bottleneck.

In recent years, ternary content addressable memory (TCAM) has been widely used for the SDN flow table, largely due to its ternary representation (0, 1,*), and the fast lookup property is quite suitable for network address matching. It is able to perform a full flow table lookup in a clock cycle in spite of the flow table size, which is at least six times faster than SRAM-based solutions. A TCAM-based flow table could be seen as an ordered array with parallel look-up abilities [4]. Entries are stored in the TCAM from high to low physical addresses in a considerably designed order; entries at higher addresses have higher matching priorities than those at lower addresses. As the matching fields of flow entries may overlap, an incoming packet will match multiple entries. In this case, only the entry at the highest physical address is chosen [5]. Thus, switches have to maintain the desired ordering of entries during flow table updates, which is important to ensure correct packet matching.

The order of entries in flow tables is determined by relationships among forwarding entries. The overlapping matching fields of entries produce dependency relationships. For instance, flow entry A: dst=192.168.1.10/32, priority=2, action=output:1 has an intersecting destination matching field with flow entry B: dst=192.168.1.0/24, priority=1, action=output:2, but the priority of *A* is higher than *B*. Thus, packets matching entry *A* will ignore *B*. In other words, entry *B* depends on entry *A*. To effectively maintain dependencies among entries, there are two prevalent approaches: integer priority and dependency graph. The integer priority value provides poor clues about rule dependencies; it brings in plenty of non-existent dependencies and oftentimes makes avoidable priority updates necessary [6]. It leads to massive redundant TCAM moves [7]. The dependency graph approach constructs a directed acyclic graph (DAG) with dependency relationships among flow entries, where nodes are the entries and directed edges represent the dependencies between adjacent nodes. With the dependency graph, we can clearly identify dependencies among entries, and focus on these actual dependencies during flow table updates. A topological sort of DAG is able to maintain the correct ordering of entries. The actual dependencies could be further utilized to help avoid unnecessary TCAM update overhead.

Hardware flow tables usually contain less than 5000 flow entries [8]. With frequent changes caused by limited table sizes, the time cost for the TCAM update is a non-linear function of the number of rules. It takes about 6 s to install 500 rules, while 1500 rules take almost 2 min [9]. An entry update takes longer when removing an existing entry and installing a new entry; thus, one update takes up to 50 ms in a 1k-sized flow table [7]. To efficiently insert new entries, existing solutions usually move existing entries to other locations to make space for new entries, considering the dependency relationship. A naive solution would shift the downstream or upstream entries next to the desired inserting location (downward or upward, each by one location), creating an empty space to store the new entry without violating the dependency relationship. The worst-case time complexity is O(N) [10]. To avoid the overhead of naive moving, an entry could be moved to a downward or upward location more freely as long as dependency is maintained. RuleTris [7] considers the dependencies among entries and moves either an upstream moving chain upward or a downstream moving chain downward. However, without fully leveraging the dependency relationships, this is not the minimum moving scheme. We further reduce the moving steps in this paper.

In this paper, we first show that a unidirectional (either upstream or downstream) moving approach can overlook chances to move downstream entries up or upstream entries down, failing to find a shorter moving chain. Moreover, inserting a new entry may introduce extra dependency relationships among existing entries, such that existing entries in the flow table may violate the new dependencies, even if they comply with previous dependencies. We propose an algorithm for single-entry updating that utilizes bidirectional moving and we prove its efficiency in minimizing update costs. Further, we design a heuristic algorithm to update the TCAM-based flow table dynamically, which supports updating multiple entries concurrently. The evaluation results show that our updating algorithms consistently outperform existing updating approaches, reducing the moving costs of TCAM entries by 6% compared to unidirectional approaches.

The rest of this paper is organized as follows: We formulate the TCAM-based flow table update problem in Section 3. In Section 4, we describe the problem of updating with a moving chain. We introduce algorithms to minimize the TCAM update overhead for inserting a single entry in Section 5 and update multiple entries dynamically in Section 6. In Section 7, we evaluate the proposed algorithms and analyze the evaluation results. Finally, we present our conclusions in Section 8.

## 2. Related Work

**Forwarding rules dependency.** In recent years, many research studies have focused on resolving the dependencies among forwarding rules, e.g., [3,11,12,13]. FLIP [14] proposes an algorithm for SDN network updates that preserves forwarding policies. It keeps track of alternative constraints, preventing repeated policy violations. However, priorities do not actually mean dependency relationships, resulting in many fake dependencies. CoVisor [15] assigns priority values in composing multiple policies of controllers without changing the priorities of existing rules, minimizing updates on existing entries. However, it is unable to resolve dependencies among rules on switches locally when switches update autonomously or when people manually configure the flow tables.

**Flow entry installation.** Flow entry installation could be divided into reactive and proactive approaches based on the first packet of a new flow arriving before or after the corresponding entry installation. As it is not possible to install all the rules in advance, reactive approaches are used to handle fine-grained traffic control in most cases, e.g., [16,17]. Once a matching failure occurs, it will take longer to establish new entries along the forwarding path, which would lead to further congestion and packet loss. Proactive approaches, e.g., [18,19,20], help reduce packet-forwarding latency with traffic prediction and estimation. However, it limits the controller’s ability to dynamically react to changes in the network traffic [21], and it does not alleviate issues related to new entry establishment latency.

**Flow table caching.** Many research studies aim to use less TCAM space to forward the vast majority of traffic; this includes dependencies among rules, e.g., [22,23]. Devoflow [2] proposes sampling and trigger techniques to figure out elephant flows, deploying flow entries only for these elephant flows. CRAFT [24] reduces the occurrence of multiple overlapping rules in the cache. Ref. [25] proposes a cover-set approach to solve the rule dependency issue, prioritizing important rules for TCAM caching. CacheFlow [26] caches small groups of rules by breaking long dependency chains while preserving the dependencies among policies. Ref. [27] designs traffic-aware architecture, monitoring the temporal behavior of rules to update flow table caching in a timely manner.

**Verify and synthesize flow table.** Automated tools verify and synthesize consistent flow tables and are also quite popular in network updating. Snowcap [28] leverages counter-examples to synthesize network configuration updates. NetStack [29] utilizes the Stackelberg game to ensure fundamental forwarding properties, such as reachability, loop freedom, and waypoint. Kaki [30] presents a Petri game-based synthesis approach. However, guaranteeing real-time update efficiency with complicated dependencies remains a challenge for automatic verification and the synthesis approach.

## 3. TCAM Maintenance Problem

Before updating the TCAM-based flow table, we need to sort the overlapping entries, so that those with longer prefixes are always situated in the top positions, with higher priority. As flow table lookups are top-down, it means that entries with higher priority are placed closer to the top of the flow table. The key notations are listed in Table 1.

**Definition** **1.** *We use $r_{i}\to r_{j}$ to denote the dependency between rule $r_{i}$ and $r_{j}$, if the matching fields of $r_{i}$ and $r_{j}$ overlap $r_{i}.match\cap r_{j}.match\neq ⌀$, and the priority of $r_{i}$ is lower than $r_{j}$$r_{i}.priority<r_{j}.priority$.*

When we update a flow table, we first have to discover dependency among entries. We can organize the dependency relationships among the rules into a directed acyclic graph G={R,E}, $R=\{r_{1},\;r_{2},\;\ldots ,\;r_{m}\}$. A function τ maps the rules to TCAM, complying with the dependency relationships.

**Definition** **2.** *TCAM-based flow table mapping is a bijective function: τ:rule→slot maps a rule to a flow table slot, and r:slot→rule obtains an entry from its position.*

Mapping rules into the TCAM-based flow table could be viewed as searching for a topological order of a dependency graph. However, there are usually many topological ordering sequences complying with the dependency relationships, resulting in different update overheads.

**Figure 1:**
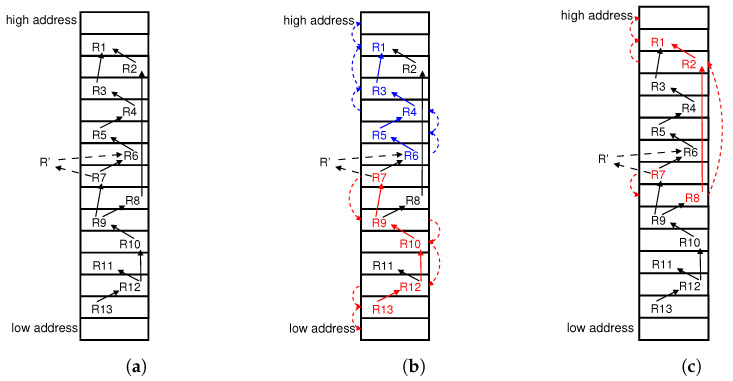
Insert a new flow entry into the existing TCAM-based flow table with (**a**) R7 depending on $R^{\prime }$ and $R^{\prime }$ depending on R6. (**b**) Moves the upstream chain R6→R5→R4→R3→R1 upward or the downstream chain R7←R9←R10←R12←R13 downward, and the minimum moving cost is 5 entries for both directions. (**c**) Checks the minimum moving chain; potentially moves each rule both upward and downward, i.g., R7, R8→R2→R1, and the minimum moving cost is 4 entries.

During the update process, the flow table may move some existing entries away to insert new rules due to the dependency relationships. To record the moving changes, we add the new rules and their dependency relationships into the directed graph, evolving from *G* to $G^{\prime }$. For example, in Figure 1a, when we insert entry $R^{\prime }$ with dependency relationships $R^{\prime }\to R6$ and $R7\to R^{\prime }$, the position of $R^{\prime }$ in the table should be between R6 and R7, although there is no empty slot to admit $R^{\prime }$ in the current mapping τ. Thus, we have to move some rules away to vacate an available slot for $R^{\prime }$. It means we need another mapping function $\tau^{\prime }$, which allows $R^{\prime }$ to be inserted into the flow table complying with the new dependency graph $G^{\prime }$ and the existing *G*.

(1) $$\begin{array}{c} \min\sum_{r\in R^{\prime }}c\left(r\right)\\ s.t.\;c\left(r\right)=\left\{\begin{array}{c} 1\;\tau^{\prime }\left(r\right)\neq \tau \left(r\right)\\ 0\;\tau^{\prime }\left(r\right)=\tau \left(r\right) \end{array}\;\forall r\in R^{\prime }\right.\\ \forall r_{i}\to r_{j}\in G^{\prime }\;\tau^{\prime }\left(r_{j}\right)>\tau^{\prime }\left(r_{i}\right) \end{array}$$

To minimize the potential update overhead while maintaining dependencies in *G* and $G^{\prime }$, we formulate the TCAM-based flow table update problem as follows. In the formulation, we attempt to minimize the number of moving rules during the updates. c(r)=1 means that the position of rule *r* changes after updating, while the dependencies are consistent with τ and $\tau^{\prime }$. The model is an integer programming (IP) problem, which is known as HP-hard.

## 4. Update Flow Table with a Moving Chain

Intuitively, inserting a new entry involves exchanging existing entries with an empty slot to make space for the inserted entry. In Figure 1a, a new rule $R^{\prime }$ is to be inserted with dependency $R7\to R^{\prime }$ and $R^{\prime }\to R6$. To avoid unexpected moves, RuleTris [7] proposes an algorithm to find a moving chain that consists of dependent rules and unrelated rules. A moving chain is a moving sequence $\left\{r_{0},\;r_{1},\;\ldots ,\;r_{k}\right\}$, where entry $r_{i}$ could be moved to replace $r_{i+1}$’s slot (i=0, 1, …, k−1), and the moving of entries along the chain complies with the dependency relationships. The length of the moving chain is the number of moving rules.

As Figure 1b shows, the shortest moving chain with RuleTris would be R7→R9→R10→R12→R13 or R6→R5→R4→R3→R1, both with five moves. However, it only considers moving either the upstream entries upward or downstream entries downward. Some entries at higher positions do not have any relationship with the insert rule, and they could be moved to lower positions. Similarly, the independent entries at lower positions may also be moved to higher positions to make space for the insertion. With this in mind, in Figure 1c, we find a shorter moving chain R7→R8→R2→R1 with only four moves, which means moving unrelated rules upward or downward may lead to fewer moving steps.

**Figure 2:**
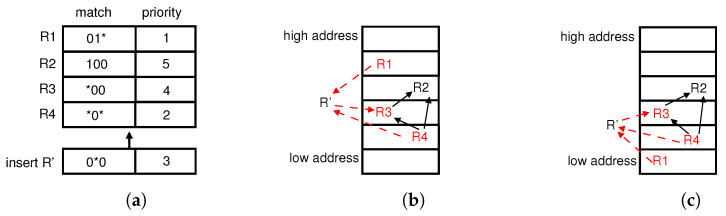
Reorder existing entries for an insert rule: (**a**) Insert $R^{\prime }$ with priority 3 into the TCAM table. (**b**) However, inserting $R^{\prime }$ directly between predecessor R1 at a lower location and successor R3 at a higher location may lead to incorrect matching. (**c**) Reorder R1, R3, R4 to insert $R^{\prime }$ between R3 and R4.

Moreover, it may be infeasible to find a moving chain with RuleTris [7]. In Figure 2a, we insert a new entry $R^{\prime }$ with matching field 0∗0, with priority 3. The matching field of $R^{\prime }$ intersects with R1(01∗), R3(∗00), and R4(∗0∗). Considering the priorities, $R^{\prime }$ depends on R3 (i.e., $R^{\prime }\to R3$) while R1, R4 depend on $R^{\prime }$ (i.e., $R1\to R^{\prime },\;R4\to R^{\prime }$). The dependencies of $R^{\prime }$ with existing entries are shown in Figure 2b. We would insert $R^{\prime }$ into the table above all the predecessors (R1, R4) and below all the successors (R3); however, there is no intersecting area. As R1 and R3 have no dependency relationship, R1 will not be swapped to a location below R3, R4 with the moving chain constructed by RuleTris [7]. Thus, we have to arrange the disorder area [R1, R2, R3] to be [R2, R3, R1] in Figure 2c before searching for a moving chain. We designed Algorithm 1 to reschedule the disorder area to make sure predecessors are always below successors. Lines 5–10 either move predecessors (that were previously placed above successors) downward or shift successors (that were formerly below their predecessors) upward. Line 7 moves the rule with the minimum moving chain heuristically until satisfying the ordering requirement of predecessors and successors. Therefore, we need to design an algorithm to search for a moving chain, which is described in Section 5.
**Algorithm 1** Reschedule existing rules for reversed dependency.1:below = $\min_{<r_{insert},r>\in E}\tau \left(r\right)$ #the lowest position of rules that $r_{insert}$ depends on2:above = $\max_{<r,r_{insert}>\in E}\tau \left(r\right)$ #the highest position of rules that depends on $r_{insert}$3:rvssucc = $\left\{r|\tau \left(r\right)<above,<r_{insert},r>\in E\right\}$ #rules that $r_{insert}$ depends on but has low address4:rvspre = $\left\{r|\tau \left(r\right)>below,<r,r_{insert}>\in E\right\}$ #rules that depends on $r_{insert}$ but has high address5:**while** below<above **do**6:   calculate chains of moving r∈rvspreto(,below], r∈rvssuccto[above,) with Algorithm 27:   r=min{length(r.chain)|r∈rvspre,rvssucc}8:   move *r* with moving chain9:   update below, above, rvssucc, rvspre10:**end while**

**Algorithm 2** TCAM update move for a single entry.
1:

chain=[]

2:**if** $\exists t,\tau \left(pre\left(r_{insert}\right)\right)<t<\tau \left(succ\left(r_{insert}\right)\right),r\left(t\right)=null$ **then**3:   $chain=[r_{insert},t]$4:
**else**
5:   **for** $t\in [\tau_{pre},\tau_{succ}]$ **do**6:     **if** $t\in [\tau \left(pre\left(r_{insert}\right)\right),\tau \left(succ\left(r_{insert}\right)\right)]$ **then**7:        $t.moves=1,t.prev=r_{insert}$8:     **else**9:        t.moves=MAX,t.prev=NULL10:     **end if**11:   **end for**12:   start $=(\tau \left(succ\left(r_{insert}\right)\right)+\tau \left(pre\left(r_{insert}\right)\right))/2$13:   offset =014:   **while** (start − offset $\geq \tau_{pre}$) or (start + offset $\leq \tau_{succ}$) **do**15:     $t_{1}$ = start − offset, $t_{2}$ = start + offset16:     **for** t = $\left\{t_{1},t_{2}\right\}$ **do**17:        **if** t $\geq \tau_{pre}$& t $\leq \tau_{succ}$&r(t)≠⌀ **then**18:            movedown = $\max_{<r,r(t)>\in G}\tau \left(r\right)$19:            moveup = $\min_{<r(t),r>\in G}\tau \left(r\right)$20:            **for** t’ ∈ [movedown, moveup] **do**21:                $t^{\prime }.prev=t^{\prime }.moves>\left(t.moves+1\right)?t:t^{\prime }.prev$22:                $t^{\prime }.moves=\min\left\{t^{\prime }.moves,t.moves+1\right\}$23:            **end for**24:        **end if**25:     **end for**26:     offset = offset + 127:   **end while**28:   $nextslot=\min_{\tau_{succ},\tau_{insert}}\left\{\tau_{succ}.moves,\tau_{insert}.moves\right\}$29:   **while** nextslot **do**30:     chain=chain.append(nextslot.prev)31:     nextslot=nextslot.prev32:   **end while**33:
**end if**
34:**return** chain


## 5. Minimizing the TCAM Update Overhead of a Single Entry Insertion

We propose an algorithm to calculate a minimum moving chain for inserting a single entry, and help reschedule disorder rules in Algorithm 1. First, we define the feasible moving range for each entry in Lemma 1, where an entry can be moved to any slot in a manner that respects dependencies. Then, we design Algorithm 2 and prove its minimum moving steps with Theorem 1.

**Lemma** **1.** *$succ\left(r_{insert}\right)=\min_{<r_{insert},r>\in E^{\prime }}\tau \left(r\right)$ is the entry that $r_{insert}$ depends on at the lowest position, and $pre\left(r_{insert}\right)=\max_{<r,r_{insert}>\in E^{\prime }}\tau \left(r\right)$ is the entry that depends on $r_{insert}$ at the highest position. $\tau_{succ}$ is the closest empty slot to $\tau (succ\left(r_{insert}\right))$, and $\tau_{pre}$ is the closest empty slot to $\tau (pre\left(r_{insert}\right))$. For all entries, $\tau \left(r\right)<\tau_{pre}$ and $\tau \left(r\right)>\tau_{succ}$, there exists a mapping $\tau^{\prime }$, satisfying $\tau^{\prime }\left(r\right)=\tau \left(r\right)$ after inserting $r_{insert}$.*

**Proof.**  If there is an empty slot $\tau \left(pre\left(r_{insert}\right)\right)<\tau_{empty}<\tau \left(succ\left(r_{insert}\right)\right)$, we can insert $r_{insert}$ into $\tau_{empty}$ without moving any other entries.

Otherwise, $\tau_{pre}<\tau \left(pre\left(r_{insert}\right)\right)<\tau \left(succ\left(r_{insert}\right)\right)<\tau_{succ}$. For $r_{insert}$ and *n* entries between $\tau_{pre}$ and $\tau_{succ}$, as $G^{\prime }$ is acyclic, the subgraph consisting of these n+1 entries $r_{insert}$ and $\forall r,\tau_{pre}<\tau \left(r\right)<\tau_{succ}$ is a directed acyclic graph. We can always find a topological order for these n+1 entries and adopt them into n+2 slots between $\tau_{pre}$ and $\tau_{succ}$. In the sub-table from $\tau_{pre}$, downward, the subgraph consisting of entries $\forall r,\tau \left(r\right)<\tau_{pre}$ below $\tau_{pre}$ does not change after the insertion, so that $\tau^{\prime }\left(r\right)=\tau \left(r\right)$. Analogously, for $\forall r,\tau \left(r\right)>\tau_{succ}$, we can also obtain $\tau^{\prime }\left(r\right)=\tau \left(r\right)$.    □

Lemma 1 indicates that we can make space for the new entry with the nearest upstream and downstream empty slots. Hence, we restrict the moving range of the rule *r* to be $\left[\tau_{pre},\tau_{succ}\right]$. With the definitions of succ(·) and pre(·), each entry *r* has no dependency with rules in slots [τ(pre(r))+1,τ(succ(r))−1]. Thus, *r* could be moved to any slot in [pre(r),succ(r)] by filling in an empty slot or replacing an existing entry, while the dependencies among entries are still maintained.

We designed Algorithm 2 to find a valid inserting update plan to insert a single entry by moving entries within $\left[\tau_{pre},\tau_{succ}\right]$, and further prove that the moving plan is the minimum. With Lines 2 and 3, if there is an empty slot between $r_{insert}$’s predecessor $pre(r_{insert})$ at the highest position and successor $succ(r_{insert})$ at the lowest position, we can insert $r_{insert}$ directly into the empty slot with chain $\left[r_{insert},t\right]$. Otherwise, we have to search for the moving chain between the nearest upstream and downstream empty slots $\left[\tau_{pre},\tau_{succ}\right]$ with Lines 5–32. We consider the potential moving steps of each entry in this region. We start searching from the middle of the region (Line 12), and then check slots one by one, until $\tau_{pre}$, $\tau_{succ}$ (Line 15). For rule r(t) in slot *t*, we check the moving step of its dependent rules in slot $t^{\prime }$, and update $t^{\prime }.moves$ and $t^{\prime }.prev$ if the moving step is larger than moving r(t) to $t^{\prime }$ (Lines 20–22). Gradually, we obtain the minimum moving step for each slot in the region. Finally, we obtain the moving chain by tracking .prev from the empty slot (Lines 28–32).

**Theorem** **1.** *The moving chain found by Algorithm 2 within $\left[\tau_{pre},\tau_{succ}\right]$ is a minimum moving chain for an entry insertion.*

**Proof.**  We construct a directed graph $DG=\{\left(r,r\left(k\right)\right)|k\in \left[\tau (pre(r)),\tau (succ(r))\right]\}$, which consists of existing entries in *G*. The edges in the graph indicate that an entry can move from the head node to the tail node while maintaining the dependencies. Algorithm 2 searches for the chain, which is the shortest path from $r_{insert}$ to the empty slot $\tau_{succ}$, or $\tau_{pre}$ with Dijkstra’s algorithm, which was proven to be the shortest in [31].

If Algorithm 2 is not a minimum-moving chain, there must be another $chain^{\prime }$ shorter than chain. We then restrict the moving range of *r* with the nearest upstream and downstream empty slots, $\tau_{pre}$ and $\tau_{succ}$, with Line 14. In the above constructed directed graph DG, the shortest path calculated by Dijkstra’s algorithm from $r_{insert}$ to *r* is $d(r_{insert},r)$, and the shortest path from *r* to an empty slot is d(r), so that the shortest path from $r_{insert}$ to an empty slot $d\left(r_{insert}\right)=\min\left\{d\left(r_{insert},r\right)+d\left(r\right)\right\}$. For $\min_{<r,r_{i}>\in E^{\prime }}\tau (r_{i})>\tau_{succ}$ or $\min_{<r_{j},r>\in E^{\prime }}\tau (r_{j})<\tau_{pre}$, if *r* is moved to $\tau_{succ}$ or $\tau_{pre}$, the shortest path from *r* to an empty slot is $d_{1}\left(r\right)=1$. Otherwise, *r* is moved to $r^{\prime }\in \left[\tau_{succ},\min_{<r,r_{i}>\in E^{\prime }}\tau (r_{i})\right]$∪$\left[\max_{<r_{j},r>\in E^{\prime }}\tau (r_{j}),\tau_{pre}\right]$, and the shortest path from *r* to an empty slot is $d_{2}\left(r\right)=d\left(r^{\prime }\right)+1$. As $d(r^{\prime })⩾0$, $d_{1}\left(r\right)⩽d_{2}\left(r\right)$, such that *r* will be moved within the range of $\left[\tau_{pre},\tau_{succ}\right]$. Iteratively, for any entry $r^{\prime }$ to be moved, $r^{\prime }$ will be moved within the range of $\left[\tau_{pre},\tau_{succ}\right]$ in each moving step. Thus, the moving chain we find within the range of $\left[\tau_{pre},\tau_{succ}\right]$ is, globally, the shortest.    □

**Complexity analysis.** The moving overhead of the entry update depends on the length of the shortest moving chain. If *c* is the diameter of the dependency graph, which is the longest shortest path between any two nodes in the graph, the moving complexity of a single entry update is less than O(c), while the computation complexity is O(N·c).

## 6. Dynamic TCAM Update for Concurrent Rules

**Figure 3:**
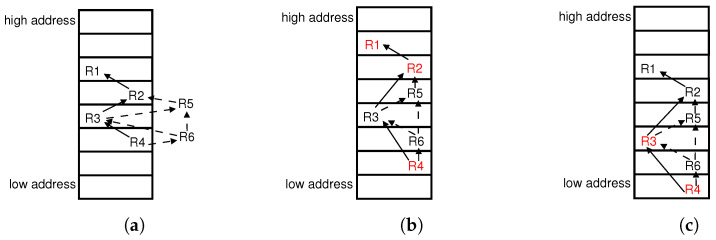
Insert multiple flow entries R5 and R6 into TCAM in (**a**). There are two insertion plans: (**b**) relocating 3 entries, R1, R2, R4, and (**c**) moving 2 entries, R3 and R4.

Multiple flows may arrive in a switch at almost the same time, or a switch may receive a batch of flow table configurations; both require updating multiple entries in one update operation. However, for the concurrent updating of multiple entries, different insertion plans may lead to diverse update overheads. For example, we insert R5 and R6 simultaneously in Figure 3. The two shortest moving chains for R5, i.g., [R1,R2] and [R4,R6,R3], both move two existing entries. The insertion plans both use the shortest moving chains for R5,R6 in Figure 3b,c, and the flow tables after updating both comply with the dependency graph. However, the overheads of the two moving plans differ. There are three existing entries relocated in Figure 3b, while two existing entries are moved in Figure 3c.

The shortest moving chains of multiple insertion entries may interact with each other by containing common existing entries (e.g., R4 in the shortest moving chains of R5 and R6) or another inserting entry (e.g., R6 in R5’s shortest moving chain [R4,R6,R3]). In Figure 3c, we move each entry in the moving chain [R4,R3] one slot downward to insert R5, while R6 further requires moving R4 downward to obtain another empty slot.

To find the appropriate empty slots for rule insertion, we designed Algorithm 3 to search the update plans for multiple entry updates. We adopt a divide-and-conquer approach to speed up the updating process. With Theorem 1, we are able to divide a flow table into multiple independent regions, and each region could be updated freely without interfering with others.

1. Divide the flow table into independent regions. The flow table could be divided into several independent insertion regions for multiple entry updates, and each insertion region could update entries independently without interfering with each other. An insertion region is a series of slots $\left[t_{a},t_{b}\right]$ in the TCAM-based flow table. The entries to be inserted in this region are $R\left(\left[t_{a},t_{b}\right]\right)=\left\{r|t_{a}⩽\tau^{\prime }\left(r_{pre}\right)⩽\tau^{\prime }\left(r\right)⩽\tau^{\prime }\left(r_{succ}\right)⩽t_{b}\right\}$. Lines 1–20 divide TCAM tables into independent regions by checking the moving range $\left[\tau_{pre}\left(r\right),\tau_{succ}\left(r\right)\right]$ of each insert rule *r*. If the moving range of *r* overlays with any other insert rule, they will be merged into a region (Lines 4–8). Merging moving ranges may cause the number of available empty slots to be less than the number of insert rules. In this case, we expand the size of the region by extending an empty slot upward and downward, respectively (Lines 10–12). Finally, we merge the regions to ensure that there is no overlap among independent regions (Line 20).
2. Update entries within each region. For each region $R(\left[t_{a},t_{b}\right])$, as we have reserved enough empty slots during the region-dividing phase, rules could be inserted into the region without interfering with other regions. We use Algorithm 2 to calculate the shortest moving chain for each rule in $R(\left[t_{a},t_{b}\right])$, and update rules in the increasing length of moving chains heuristically (Line 22).

**Algorithm 3** Dynamic TCAM update.
1:Regions={} #Regions contains a set of moving regions2:**for** each $r\in R_{insert}$ **do**3:   calculate $\tau_{pre}\left(r\right)$ and $\tau_{succ}\left(r\right)$4:   **if** $\exists rgn\in Regions,\left[rgn.start,rgn.end\right]\cap \left[\tau_{pre}\left(r\right),\tau_{succ}\left(r\right)\right]\neq ⌀$ **then**5:       #update an existing region6:       rgn.insert=rgn.insert∪r7:       $rgn.start=\max(rgn.start,\tau_{pre}\left(r\right))$8:       $rgn.end=\min(rgn.end,\tau_{succ}\left(r\right))$9:       #expand rgn until containing enough empty slots to accommodate rules rgn.insert10:       **if** $\left|\{t|r\left(t\right)=⌀,t\in \left[rgn.start,rgn.end\right]\}|<|rgn.insert\right|$ **then**11:          rgn.start=max{t|r(t)=⌀,t<rgn.start}12:          rgn.end=min{t|r(t)=⌀,t>rgn.end}13:       **end if**14:   **else**15:       #create a new region16:       rgn.insert={r}17:       $rgn.start=\tau_{pre}\left(r\right),rgn.end=\tau_{succ}\left(r\right)$18:   **end if**19:
**end for**
20:merge rgns in Regions until there is no intersection21:**for** each rgn∈Regions **do**22:   update rgn.insert in increasing length of chain calculated by Algorithm 223:
**end for**



**Complexity analysis.** With Algorithm 2, the computation complexity of Algorithm 3 is O(k·N·c), where *k* is the number of concurrent entries. As the time cost and power consumption of the TCAM entry updates are much higher than the computation cost, we prefer to exchange the computational cost for enhanced hardware efficiency.

## 7. Evaluation

To evaluate the effectiveness of our proposed algorithms, we conduct various experiments with different dependency complexities. As the dependencies among rules are constructed as directed acyclic graphs, we generate dependency graphs with different connectivity and shapes. We evaluate two kinds of dependency complexities by varying: (1) dependency graph density, i.e., forwarding rules with more complex dependencies form a dense graph, while fewer dependencies result in a sparse graph. We adjust the densities of dependency graphs by setting the node degrees *d*. By changing *d*, we can generate three kinds of graph densities: a sparse graph with a small *d* value (Sparse), a medium complex graph with a moderate *d* value (Medcplx), and a dense graph with a big *d* value (Dense). (2) Dependency graph shape: Rules with longer dependency chains form a slim but long dependency graph, while rules with shorter dependency chains form a fat but short dependency graph. We adjust the shape of the dependency graph by controlling the ratio of the width to length. During the graph construction, we put all the nodes with k predecessors in the longest dependency chain into the k-th layer, so that we obtain a dependency graph with *l* layers. We treat *l* as the length of the graph, and the maximum number of nodes *w* in each layer as the width of the graph. By varying *l* and *w*, we are able to generate three kinds of graph shapes: slim and long graphs with small w/l (<0.5) ratios (SimLong), medium-shaped graphs with middle w/l (0.5∼1.5) ratios (Medium), fat and short graphs with big w/l (>1.5) ratios (FatShort). We simulate a TCAM-based flow table, where the capacity is 10,000 entries, and we generate different dependency graphs by adjusting the graph‘s density and shape.

**Figure 4:**
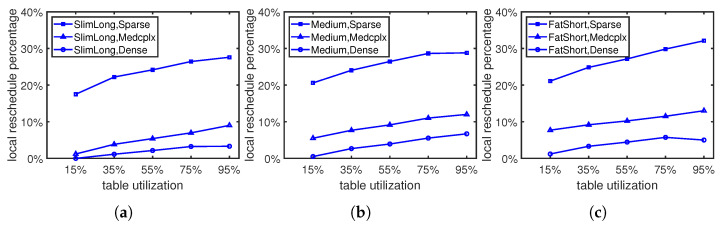
Reschedule existing rules with different dependency graph shapes: (**a**) Slim and long graph. (**b**) Medium graph. (**c**) Fat and short graph.

We first check the possibility of rescheduling existing rules under different flow table utilizations (low, med, high). Figure 4 shows that the rescheduling possibility increases with the flow table utilization, as more rules in the flow table may potentially introduce more reversed dependency during the updates. For dependency graphs with different complexities, we find that sparse graphs have higher rescheduling possibilities than dense graphs because dense graphs impose more—and complex—restrictions on dependency relationships among rules compared to sparse graphs, which are not easy to break during updates. In sparse dependency graphs, 20–30% of updates need to reschedule local rules, while the percentage of dense graphs is less than 10%. For dependency graphs with different shapes, the rescheduling possibility increases slightly as the shape of the dependency graph becomes fatter and shorter, from Figure 4a,b to Figure 4c. Entries in fat and short graphs have shorter dependency chains compared with slim and long graphs, where entries at high locations may have no relationships with those at lower locations, making entry insertions in fat and short graphs more likely to lead to reversed dependencies.

We compare our single-entry updating Algorithm 2 (Bidirect) with the existing single direction-moving approach (Sidirect), e.g., RuleTris [7] and FastRule [5], as shown in Section 7.1. Then, we evaluate our concurrent updating Algorithm 3 (denoted by H) and compare it with update entries in random order (denoted by O), which is a widely-used mechanism for multiple updates, as shown in Section 7.2.

### 7.1. A Single Entry Update Evaluation

For the single entry update in the flow table, we check the moving steps in different flow table utilizations, dependency graph shapes, and densities, respectively. Fewer moving steps indicate less update overhead, which is expected to reduce the TCAM maintenance cost and improve traffic forwarding efficiency.

**Figure 5:**
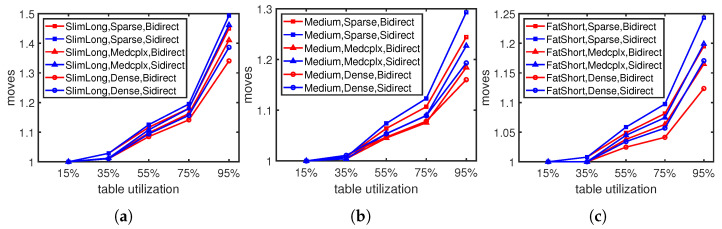
Moving steps under different flow table utilizations: (**a**) Slim and long graph. (**b**) Medium graph. (**c**) Fat and short graph.

Figure 5 shows the variation in moving steps as the flow table utilization changes under different dependency graph shapes. When the flow table utilization rises, the moving steps rapidly increase. As the higher flow table utilization leaves less available storage space, the flow table needs to move more existing rules to make space for new entries. The evaluation results show that our moving approach takes fewer moving steps compared with Sidirect in any dependency graph shape. In particular, under high flow table utilization, which means the flow table is almost full, our moving cost is 0.6–5.2% lower than Sidirect, which is the most common scenario in daily life. Fat and short graphs tend to take fewer moving steps with shorter dependency chains, as Figure 5c shows, than slim and long graphs with longer dependency chains, as shown in Figure 5a,b.

**Figure 6:**
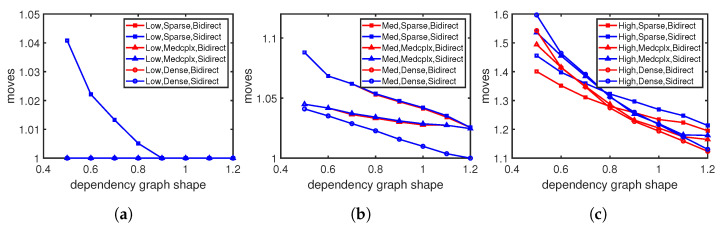
Moving steps with different dependency graph shapes: (**a**) Under low flow table utilization. (**b**) Under medium flow table utilization. (**c**) Under high flow table utilization.

Figure 6 shows that the moving steps vary with the dependency graph shapes under different flow table utilizations. When the ratio of the width to length increases, the moving steps decrease under any flow table utilization. The increasing width/length ratio indicates that the dependency graph is becoming fatter and shorter and contains more shorter dependency chains. The shorter chains take fewer moving steps to accommodate new entries. The moving steps increase as the flow table fills up, as seen in Figure 6a–c, and are in alignment with the results in Figure 5. Our moving approach consistently incurs no more cost than Sidirect, regardless of the graph shape; in particular, under high flow table utilization, our average cost is 0.7–4.9% lower than Sidirect.

**Figure 7:**
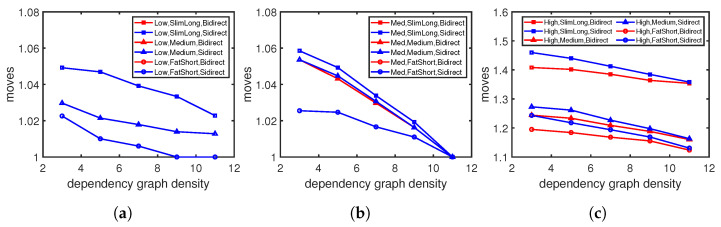
Moving steps with different dependency densities: (**a**) Low flow table utilization. (**b**) Medium flow table utilization. (**c**) High flow table utilization.

Figure 7 shows that the moving steps change with the dependency complexity under different flow table utilizations. We find that the moving steps decrease when the dependency graph density rises. Dense graphs contain more dependency relationships among entries compared with sparse graphs, which means more moving opportunities. Thus, with dense graphs, there is a higher likelihood of selecting a shorter moving chain for an entry insertion. This explains why sparse graphs consume more moving steps than dense graphs in Figure 5 and Figure 6. Similar to the results with different graph shapes, updates under high flow table utilization in Figure 7c take more moving steps than those under the lower table utilization in Figure 7a,b. The advantage of our moving approach is more significant under high flow table utilization, where our average moving cost is 1–4.9% lower than Sidirect.

### 7.2. Dynamic Updating Evaluation

To examine the dynamic update effectiveness of our moving approach, we update 1–10 entries at once in the flow table. We compare our heuristic updating Algorithm 3 (denoted as H) with updating entries, one by one, in a random sequence (O), combined with our minimum moving Algorithm 2 (B) and Sidirect (S), respectively.

**Figure 8:**
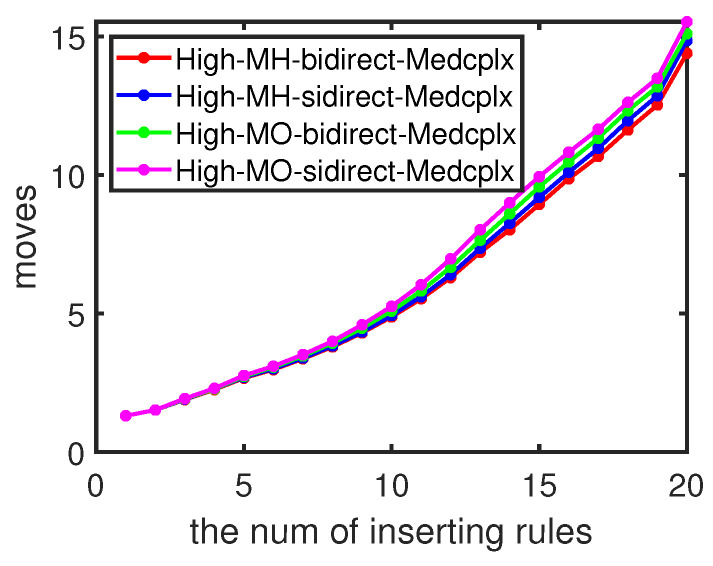
Dynamic moving with different numbers of insert rules.

**Figure 9:**
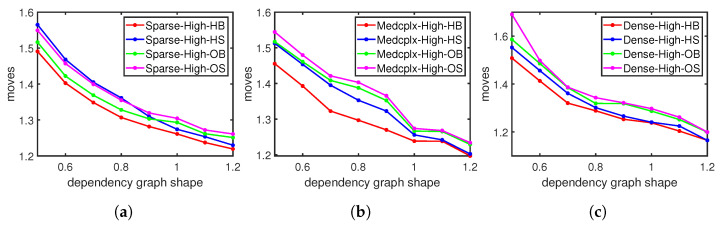
Dynamic moving with different dependency graph shapes: (**a**) Sparse-dependency graph. (**b**) Medcplx-dependency graph. (**c**) Dense-dependency graph.

We first check the updating cost with different numbers of concurrent rules. The moving steps increase rapidly when we insert more rules simultaneously in Figure 8, as more rules usually indicate more dependencies to resolve, which results in more moving steps. We then compare the performances of different approaches when we insert 1–5 concurrent rules into the flow table. Figure 9 shows the moving steps under high flow table utilization with varying graph density. The moving steps decrease as the width/length ratio increases in all three scenarios, which are consistent with the single-entry updating in Figure 6. The heuristic updating method for minimum movement (HB) consistently requires fewer steps compared to other approaches; this is more evident with sparse dependency graphs. The average cost with our heuristic updating method could be 2.1–6.4% lower than updating one by one.

**Figure 10:**
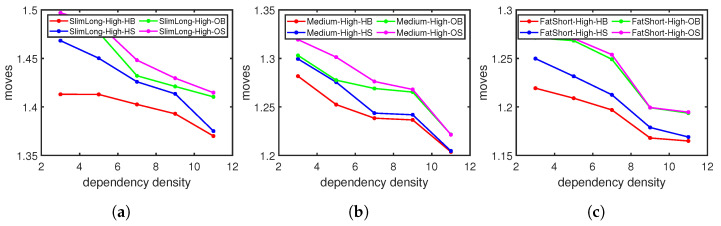
Dynamic moving steps with different dependency densities: (**a**) Slim and long graph. (**b**) Medium graph. (**c**) Fat and short graph.

Figure 10 shows the moving steps under high flow table utilization with different graph shapes. Similar to the single-entry updating in Figure 7, the moving steps decrease when the dependency density increases. Our heuristic updating method with minimum moving (HB) also achieves the lowest moving cost compared with other updating approaches; this is about 1.8–6.3% lower than updating one by one. The updates on slim and long graphs, as seen in Figure 10a, tend to take more steps with longer dependency chains than those on fatter and shorter graphs, as seen in Figure 10b,c.

## 8. Conclusions

Considering the expensive price and limited storage space of TCAM, in this paper, we focus on reducing the updating cost of the TCAM-based flow table. We first formulate the TCAM-based flow table maintenance as an NP-hard problem. To solve the problem, we propose a minimum moving algorithm for inserting a single entry, and prove its correctness. We then extend it to a heuristic algorithm to dynamically and concurrently update entries. The evaluation results with various dependency graphs show that our updating algorithm achieves an approximate 6% cost reduction compared to existing approaches for each entry updating. It means faster packet forwarding and less energy consumption. As SDN introduces more innovations in network virtualization, such as network isolation and fine-grained flow control, this generates more forwarding rules within the data plane. The proposed approach is able to help handle a large number of forwarding rule updates with enhanced efficiency. Meanwhile, real-time networks have more strict low latency requirements, especially in automotive, medical, and industry areas. The reduced update costs of TCAM-based switches are more beneficial for speeding up real-time communication efficiency.

## Figures and Tables

**Table 1:** Key notation.

Notation	Definition
τ(rule)	position of entry rule
r(slot)	entry at position slot
p(rule)	priority of entry rule
$<r_{i},r_{j}>$	edge $r_{i}\to r_{j}$ in dependency graph ($r_{i}$ depends on $r_{j}$)
